# Editorial: Germline development: From germline stem cells to gametes, Volume II

**DOI:** 10.3389/fcell.2023.1193343

**Published:** 2023-04-03

**Authors:** Myon Hee Lee, Rosa E. Navarro, Sung Min Han

**Affiliations:** ^1^ Department of Internal Medicine, Division of Hematology and Oncology, Brody School of Medicine at East Carolina University, Greenville, NC, United States; ^2^ Department of Biology, East Carolina University, Greenville, NC, United States; ^3^ Departamento de Biología Celular y Desarrollo, Instituto de Fisiología Celular, Universidad Nacional Autónoma de México, Mexico City, Mexico; ^4^ Department of Physiology and Aging, Institute on Aging, College of Medicine, University of Florida, Gainesville, FL, United States

**Keywords:** germline development, germline stem cell, differentiation, sex determination, gametogenesis

Adult stem cells have been found in a wide range of tissues throughout the body of most organisms, including humans. These adult stem cells serve to replace cells that are lost or damaged in the tissue as needed. Among the adult stem cells, germline stem cells (GSCs) have been intensively studied in both invertebrates and vertebrates with different sexes or both sexes (Spradling et al., 2011; Wu et al., 2013). In most organisms, the GSCs often reside in a special microenvironment, a GSC niche (Spradling et al., 2011). The GSC niche is a somatic cell that provides extrinsic signaling to maintain GSCs (Lander et al., 2012). Within the GSCs, intrinsic factors, including transcription factors, mRNA regulators (e.g., microRNAs), cell cycle regulators, and signaling molecules, also regulate a balance between their self-renewal and differentiation potential (Spradling et al., 2011; Wu et al., 2013).

During germline development, GSCs make a number of cell fate decisions - self-renewal/differentiation, sperm/oocyte, and apoptosis/survival decisions (Lee et al., 2020). Aberrant regulation may result in either loss of a specific cell type or uncontrolled cell proliferation, associated with infertility and germline tumors, respectively (Lee et al., 2020). Although significant progress has been made in dissecting the molecular mechanisms of each type of regulation in invertebrates and vertebrates, understanding the intricate interplay among the regulatory networks during germline development remains a major challenge.

Proliferation versus differentiation is an essential decision in the germline. Unbalance between these choices can lead to severe consequences for the organism. Therefore, many factors have evolved to control these important decisions in the germline, which is why redundancy in regulating proliferation versus differentiation exists in the organism. In this issue, Broek et al. offer a comprehending review about the regulation of germ cell proliferation versus differentiation and its redundancy in the model organism *Caenorhabditis elegans* (Vanden Broek et al., 2022).

The *Drosophila* ovary is a tube-like structure that consists of repeated units called ovarioles. Each ovariole contains a linear array of egg chambers composed of germ cells and somatic cells. The somatic cells include escort cells (ECs) located at the anterior end of the egg chamber. Chen et al. discuss the new role of canonical Wnt signaling in promoting the formation and protrusions of ECs in ovarian somatic cells (Chen et al.). These cells serve as a physical barrier for germ cells, preventing aberrant activation of morphogen signaling, such as Dpp/Wnt signaling produced by the GSC niche. ECs physically define morphogen territories and allow for proper differentiation, limiting Dpp/Wnt signaling to GSCs only. The authors demonstrate that when canonical Wnt signaling is disrupted, EC numbers decrease, and their cellular protrusions are disrupted, leading to the diffusion of morphogens to GSC progeny and suppression of germ cell differentiation. This paper is interesting, as the physical barrier created by EC protrusions may exist in other organisms and organs as determinants of tissue patterning.

While over 2,000 mutations affecting male fertility have been discovered in *Drosophila*, most remain poorly understood. The *cnt1* gene is a conserved gene that facilitates the influx of nucleosides through the transmembrane sodium gradient. The study conducted by Maaroufi et al. investigated the role of the *cnt1* gene in male fertility (Maaroufi et al.). They used the CRISPR/Cas9 system to create a mutation in the *fly cnt1* gene. The *cnt1* mutation caused defects in mating behavior and spermatid maturation, significantly reducing male fertility. Specifically, the authors report that the effects of the *cnt1* mutation are pleiotropic, including the disrupted structure of the spermatid tail, abnormal mitochondrial morphology, and a higher number of spermatid groups and mature sperms. The authors also showed that the *cnt1* mutation causes a disorganized spermatid tail structure and reduced mature sperm in the seminal vesicle. The importance of nucleoside transport in male fertility might not be limited to *Drosophila*. Cnt isoforms have also been identified in human and rat testes, particularly in Sertoli cells, which are crucial for sperm nutrition. Blocking nucleoside transporters in Sertoli cells can interfere with the spermatid maturation process, suggesting the potential importance of nucleoside transport in sperm maturation in mammals.

Fang et al. evaluated the differentiation potential for the human germline of idiopathic non-obstructive azoospermia (NOA) patient-specific induced Pluripotent Stem Cells (iPSCs) (Fang et al.). Specifically, the authors compared the differentiation potential of the iPSCs of NOA patients with normal iPSCs derived from fertile men. The results indicated that the idiopathic NOA patient-specific iPSCs exhibited decreased efficiency for early primordial germ cell (PGC) induction *in vitro* and defects in the expression of key genes involved in PGC specification. Moreover, transcriptome analysis revealed that an *in vitro* differentiation potential might be correlated with the apoptosis mechanism. These findings may provide insights for future studies on the mechanism of male infertility.

Efforts to elucidate heterogeneity in adult stem cell populations are critical for advancing our understanding of how these populations change over time and in response to niche perturbations. Stolzenbach et al. propose that GSC heterogeneity is increased with age or under stress with evidence from mammalian systems and with two different kinds of adult stem cells–hematopoietic stem cells and GSCs (Stolzenbach et al.). Based on the research findings, the authors further propose that clonal selection may be prevalent in the GSCs during aging. The authors also highlighted the need for additional research on microenvironmental pressures, clonal heterogeneity, and non-neutral clonal selection in stem cell populations. Those may provide new opportunities for detecting and treating congenital abnormalities, disease initiation, and aging-associated pathologies.

Spermatogonial stem cells (SSCs) provide the basis for the life-long production of enormous numbers of sperm. Culture mouse SSCs can keep their spermatogenic potential and epigenetic stability for up to 2 years. SSCs culture offers the potential for editing genes to test their function and understand spermatogenesis and the causes of infertility. There are some alternatives for specific editing of the genome. Still, CRISPR/Cas9 reaches higher and comparable editing rates while keeping SSCs differentiation *in vivo* and producing healthy non-mosaic offspring carrying the desired genetic modification. In this issue, Obermeier et al. published a new protocol for gene editing in male germline stem cells, which shows a potent tool to study spermatogenesis and to create transgenic mice (Obermeier et al.). The difference with other protocols is that this method uses lipofection of site-specific Cas:gRNA ribonucleoprotein complexes to target sites *via* non-homologous end joining. Among the advantages of this method is that it saves costs and is technically less complex when compared to others. It is also non-integrative, produces fewer off-target effects, and can be used for large loss-of-function studies. A significant advantage of this protocol is that germline stem cells maintained their spermatogonial potential.

In summary, the results reported in this research topic may provide key insights into GSC maintenance and its differentiation into sperm or oocytes in invertebrate and vertebrate model systems. Furthermore, insights gained from GSC studies are invaluable in the directed differentiation of other adult stem cells. Therefore, a better understanding of all the regulators and mechanisms involved in the maintenance and differentiation of GSCs will lead us to novel and innovative approaches, which form the basis of cell therapy and tissue engineering targeted toward multiple stem cell-associated diseases.
